# Effect of Fenton-Based Processes on Arsenic Removal in the Presence of Humic Acid

**DOI:** 10.3390/toxics12120845

**Published:** 2024-11-25

**Authors:** Wenming Xiong, Qixuan Huang, Langlang Li, Yongjun Li

**Affiliations:** 1 Guangzhou Vocational College of Technology & Business, Guangzhou 511442, China; xiongwenming123@163.com; 2 Guangdong Laboratory for Lingnan Modern Agriculture, Guangdong Provincial Key Laboratory of Agricultural & Rural Pollution Abatement and Environmental Safety, College of Natural Resources and Environment, South China Agricultural University, Guangzhou 510642, China; 2637340848@stu.scau.edu.cn (Q.H.); lilanglang@stu.scau.edu.cn (L.L.)

**Keywords:** Fenton-based processes, arsenic removal, the presence of humic acid

## Abstract

Geogenic arsenic (As) contamination in groundwater poses a significant public health risk in many regions worldwide. Previous studies have reported hydrogen peroxide (H_2_O_2_) concentrations ranging from 5.8 to 96 μmol L^−1^ in rainwater, which may contribute to the oxidation and removal of As. However, the influence of natural organic matter, such as humic acid (HA), on rainwater-borne H_2_O_2_-induced Fenton processes for the oxidation and removal of As remains unclear. In this study, the Fenton process was employed to investigate changes in As(V), As(III), and their mixtures, both in the presence and absence of HA. The results showed that low concentrations of HA (0–10 mg/L) promoted the oxidation of As(III) and removal of As(V) when As(V) and As(III) were present individually. However, when As(V) and As(III) coexisted, HA inhibited the Fenton process for As(V) removal. This inhibition was likely due to As(III) competing strongly with HA for hydroxyl radicals in the Fenton reaction system. Additionally, the presence of HA hindered the Fe(III)-driven removal of As(V), a product of the Fenton reaction. These findings further enhance our understanding of the potential role of rainwater-borne H_2_O_2_ in the transformation of As species in open water environments.

## 1. Introduction

Arsenic (As) is a widespread contaminant in various subsurface drinking water aquifers worldwide [1,2]. Many groundwater systems have been reported to significantly exceed the safe As guideline value of 10 μg/L set by the World Health Organization (WHO) [3]. As migration and transformation in the groundwater system are influenced by complex hydrogeological conditions, geochemistry, and biological processes [4]. Furthermore, previous studies have indicated that the interface between surface water and groundwater serves as a hotspot for significant As release, characterized by a high presence of labile dissolved organic matter and As-bearing Fe(III) hydroxides [5,6,7]. The released As may further be leached into the shallow groundwater, resulting in high-As groundwater [8,9]. In addition, the As(V) in groundwater has been proved to precipitate or adsorbe onto Fe(III) oxide surfaces, thus limiting As migration and transformation [10,11]. Therefore, understanding the chemical behavior of As in water environments is essential for developing management strategies to minimize the ecological impacts of As in aquatic ecosystems.

Rainwater is commonly found to contain hydrogen peroxide (H_2_O_2_) at micromolar concentrations of 5–100 μM [12,13]. Fenton reactions, which involve the conversion of H_2_O_2_ into hydroxyl free radicals (•OH) in the presence of ferrous iron (Fe^2^⁺), are effective for oxidizing contaminants [14]. The findings from previous simulation experiments indicate that micromolar concentrations of H_2_O_2_ play a crucial role in the removal of As(III) from water by inducing a strong oxidative Fenton effect on the surface of zero-valent iron [15,16]. Earlier research has shown that rainwater-borne H_2_O_2_-driven Fenton reactions promote the oxidation of highly mobile As(III) to less mobile As(V) species, thereby significantly reducing the bioavailability of arsenic in paddy soils [11,17]. Furthermore, As(III) has been found to strongly compete with humic acid (HA) for available hydroxyl radicals in Fenton reaction systems [18,19,20,21]. However, the specific impact of natural organic matter, such as HA, on the Fenton-driven oxidation and removal of arsenic remains unclear.

Therefore, this study conducted experiments to examine the changes in arsenic species in the presence of either As(V) or As(III), individually or in combination with humic acid (HA). The objective was to gain deeper insights into the interactions between H_2_O_2_, arsenic species, and HA, thereby improving our understanding of the impact of Fenton-based processes on arsenic removal in open water environments.

## 2. Materials and Methods

### 2.1. Materials

Analytical-grade sodium arsenate (Na_2_HAsO_4_·12H_2_O, As(V)), arsenic trioxide (As_2_O_3_, As(III)), 30% purity hydrogen peroxide (H_2_O_2_), ferrous sulfate (Fe_2_SO_4_·7H_2_O, Fe^2+^), ferric sulfate hydrate (Fe_2_(SO_4_)_3_·xH_2_O, Fe^3+^), humic acid (C_9_H_9_NO_6_, HA), sodium hexane sulfonate (C_6_H_13_NaO_3_S), guaranteed reagent-grade citric acid (C_6_H_8_O_7_), and ammonia (NH_3_·H_2_O) were acquired from Guangzhou Chemical Reagent Factory (Guangzhou, China). Ultrapure water with a conductivity of 18.2 MΩ/cm was used throughout all experiments.

### 2.2. Analytical Techniques

#### 2.2.1. Sample Preparation

As_2_O_3_ was dissolved in NaOH to the required concentration. HA was pre-dissolved in 7 mM NaOH, filtered using a 0.22 μm cellulose ester membrane, and neutralized with hydrochloric acid to prepare the HA stock solution (TOC = 250 mg/L, pH = 7.0 ± 0.2), which was stored at 4 °C, away from light.

#### 2.2.2. Determination of As(III) and As(V)

As(V) and As(III) measurements were conducted using an HPLC-ICP-MS system. For HPLC (Agilent 1260) separation, an Athena C18-WP column and guard column were used. The mobile phase was a mixture of citric acid and sodium sulfonate, with a flow rate of 1.0 mL/min and an injection volume of 20 μL. For ICP-MS (Agilent 7700, Agilent Technologies, Inc., Santa Clara, CA, USA), argon was the carrier and make-up gas. Detailed instrumental operating conditions are provided in Supplementary Table S1. The concentration of HA was measured using a TOC analyzer (Vario TOC Elementar, Elementar Analysensysteme GmbH, Langenselbold, Germany). The pH was measured using a calibrated pH meter (model: SX-620), and the redox potential (Eh) was measured using a REDOX potential meter (THERMO ORION, Thermo Fisher Scientific Inc., Waltham, MA, USA).

### 2.3. Experimental Design

Experiments were conducted to investigate the behavior of arsenic species under various conditions, both with and without HA. In the absence of HA, two setups were used: a control and a Fenton reaction treatment. In the control setup, the concentrations of As(V), As(III), and As(V)/As(III) were each set to 1 mg/L, with a pH of 7.0, a temperature of 298 K, and a reaction time of 60 min. For the Fenton experiments, the same concentrations of As species were maintained, with the addition of 100 µM H_2_O_2_ and 100 µM Fe(II), under the same pH, temperature, and reaction time. In the presence of HA, both a control and a treatment were designed. The control maintained the As concentrations at 1 mg/L with varying HA concentrations (0–25 mg/L), a pH of 7.0, a temperature of 298 K, and a reaction time of 60 min. The Fenton experiments with HA followed similar conditions, with the addition of 100 µM H_2_O_2_ and 100 µM Fe(II). Additionally, Fenton-like experiments were conducted using Fe(III) products from the rainwater-borne H_2_O_2_-induced Fenton process to examine changes in As species in systems containing As(V), As(III), or their combination, in the presence of HA. These experiments were performed with As concentrations of 1 mg/L, HA concentrations ranging from 0 to 25 mg/L, 100 µM H_2_O_2_, 100 µM Fe(III), a pH of 7.0, a temperature of 298 K, and a reaction time of 60 min.

Centrifuge plastic tubes with a 10 mL capacity were utilized as batch reactors in this study. Each tube was filled with 10 mL of either As(V) or As(III) or coexisting solution at a concentration of 1 mg/L to explore the impact of different concentrations of HA on As transformation during Fenton processes. The experiments were conducted over a duration of 1 h to ensure complete consumption of •OH or H_2_O_2_. The entire experiment was conducted at room temperature (25 ± 2 °C). At the end of the experiment, solution samples were collected for the analysis of As species (As(V) and As(III)), determination of total organic carbon (TOC), pH and redox potential. These samples were subsequently stored at −20 °C prior to analysis.

### 2.4. Statistical Analysis

The experimental data were processed using Excel (version 2019), and statistical analyses were conducted with SPSS 26. An independent t-test was performed to calculate the means and standard errors for the control and Fenton treatment groups at a 5% significance level. A one-way ANOVA was used to evaluate the effect of HA-mediated Fe(III) on As species in water at different HA concentrations. Additionally, Origin 2024b software was employed to generate the graphical plots.

## 3. Results

### 3.1. Effect of HA Concentration in Fenton Process

**Figure 1:**
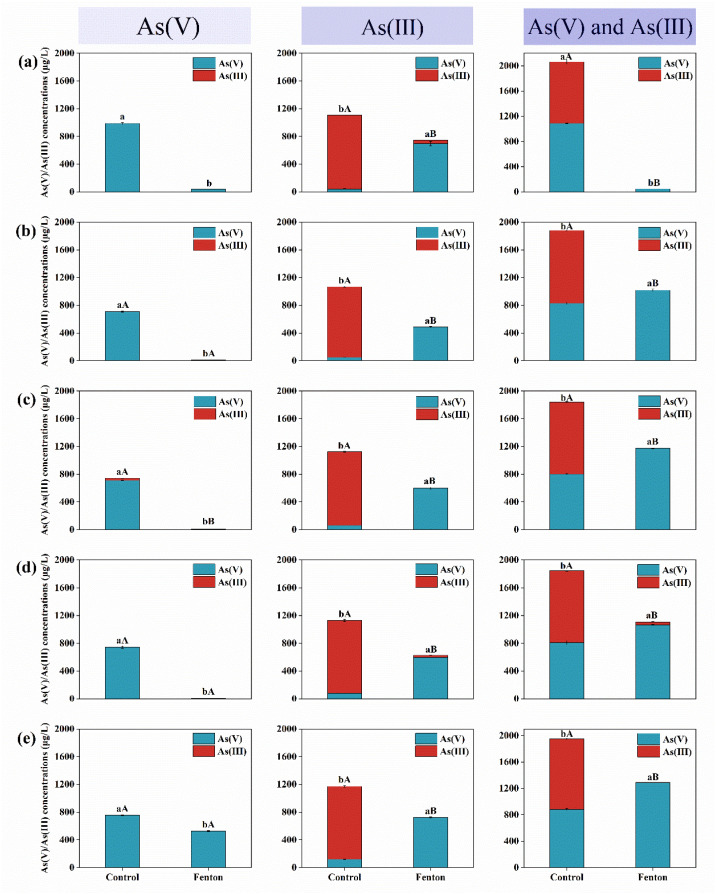
Effect of control and Fenton treatments on arsenic species in water across different humic acid (HA) concentrations: (**a**) 0 mg/L HA, (**b**) 2.5 mg/L HA, (**c**) 5 mg/L HA, (**d**) 10 mg/L HA, and (**e**) 25 mg/L HA. Different lowercase letters indicate statistically significant differences in As(V) content, while different uppercase letters denote statistically significant differences in As(III) content, both determined at *p* < 0.05.

In water contaminated with 1 mg/L As(V), the Fenton treatment resulted in a significantly lower As(V) concentration compared to the control (*p* < 0.05). In water polluted with As(III), the Fenton treatment oxidized most of the As(III) to As(V). In contrast, in the control treatment, As remained predominantly in the As(III) form, with only a small portion oxidizing. Further investigation of water containing both As(V) and As(III) showed that under Fenton conditions, most As(III) was oxidized to As(V), while only a minor fraction was oxidized in the control (Figure 1a,b).

When the HA concentration was increased to 5.0 mg/L, the removal rate of As(V) in the Fenton treatment exceeded 99% (Fenton: 99.36%). However, when As(III) was present alone or coexisted with As(V), the control treatment (without Fenton treatment) led to As(III) remaining the dominant species in the water (Figure 1c).

The results for the 10.0 mg/L HA-mediated Fenton reaction are shown in Figure 1d. The Fenton treatment significantly reduced As(V) levels, with concentrations notably lower than in the control (*p* < 0.05). In water containing only As(III), the control treatment resulted in As(V) and As(III) concentrations of 77.47 μg/L and 1053.06 μg/L, respectively. Under Fenton treatment, As(V) and As(III) concentrations were 595.93 μg/L and 30.37 μg/L, respectively. These results suggest that 10.0 mg/L HA interfered with the Fenton reaction’s ability to oxidize As(III), thereby reducing its capacity to remove As(V).

When the HA concentration was further increased to 25.0 mg/L (Figure 1e), the As(V) removal rate in the Fenton treatment dropped to below 50% (Fenton: 47.58%). However, in water containing only As(III) or a mixture of As(V) and As(III), the HA-mediated, rainwater-borne H_2_O_2_-driven Fenton reaction enhanced the oxidation of As(III).

Figure 1 shows that in water contaminated with 1 mg/L As(V) or As(III), HA at concentrations between 2.5 and 10.0 mg/L promoted As(V) removal. Furthermore, the HA-mediated, rainwater-borne H_2_O_2_-driven Fenton reaction removed significantly more As(V) than HA alone (*p* < 0.01). However, at an HA concentration of 25.0 mg/L, the Fenton reaction became less efficient at removing As(V) from water. Additionally, when As(V) and As(III) coexisted in the water, the HA-mediated Fenton reaction at concentrations between 2.5 and 25.0 mg/L showed a significantly lower removal effect on As compared to the control (*p* < 0.05).

In summary, adding HA in the 2.5 to 25.0 mg/L concentration range to natural water significantly influences effect of the rainwater-borne H_2_O_2_-driven Fenton reaction on As species. The results indicate that (1) As(V) removal efficiency is highly dependent on HA concentration, with a noticeable decline at higher levels; (2) HA enhances the Fenton reaction’s ability to oxidize As(III), though its concentration has a minimal effect on this process; and (3) when both As(V) and As(III) coexist, the HA-modified Fenton reaction is less efficient for As removal compared to individual treatments, with As(III) being more impacted.

### 3.2. Correlation Analysis of HA Concentrations and As(V) or As(III) Concentrations in Different Treatments

**Figure 2:**
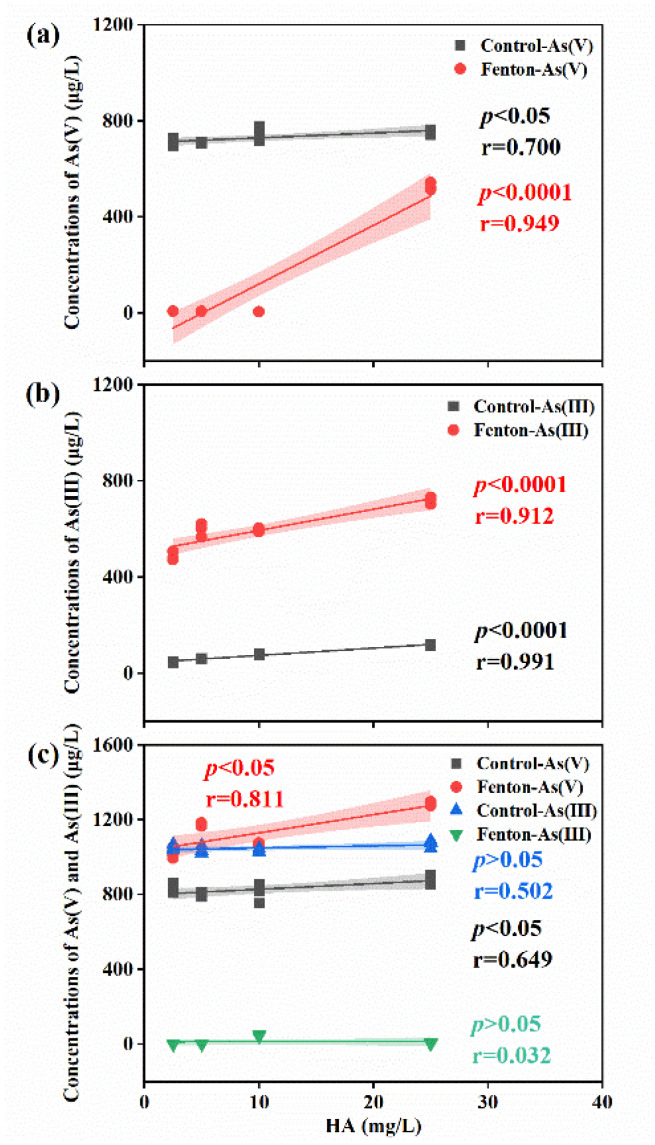
Correlation between HA concentration and As(V)/As(III) concentrations in (**a**) As(V), (**b**) As(III), and (**c**) As(V) and As(III).

In three experimental conditions—1 mg/L As(V) alone, 1 mg/L As(III) alone, and a mixed solution containing 1 mg/L of both As(III) and As(V)—the concentration of As(V) showed a significant positive correlation with the concentration of HA in both the control and Fenton treatments (*p* < 0.05 for significance, *p* < 0.0001 for extreme significance, as shown in Figure 2). These findings suggest that naturally occurring HA plays a role in the removal of As(V) from water, with the removal efficiency decreasing as HA concentrations increase, which aligns with previous research [22].

### 3.3. Effect of Fenton-like Treatment Under Different HA Concentrations

Fe(III) plays a key role in the Fenton reaction by enhancing the adsorption capacity of colloidal components through hydrolysis and facilitating the production of OH- [23]. Additionally, it promotes the interconversion between Fe(II) and Fe(III) through its catalytic activity. Ritter et al. [24] demonstrated that As(V) can use Fe(III) as a bridge to form close associations with Fe(III)-NOM colloids, which is important for the transformation of As in the environment.

**Figure 3:**
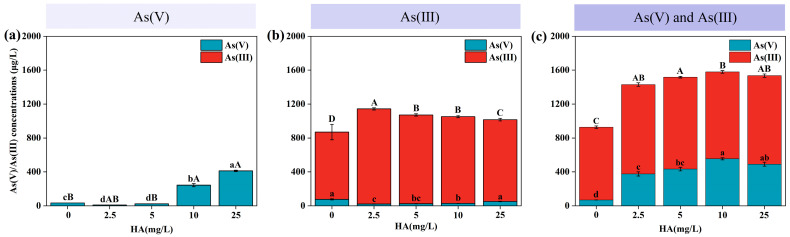
Effect of Fenton-like treatment on As species in water: (**a**) As(V), (**b**) As(III), and (**c**) As(V) and As(III). Different lowercase letters indicate significant differences in As(V) concentrations, while different uppercase letters indicate significant differences in As(III) concentrations (*p* < 0.05).

The concentration range of HA, from 2.5 to 25 mg/L, commonly found in natural water, significantly influenced the effect of Fe(III) treatment on As species in water [25]. As shown in Figure 3a, increasing HA concentrations reduced the removal of As(V) by Fe(III) treatment. Specifically, HA concentrations of 10.0 mg/L and 25.0 mg/L inhibited the Fe(III)-mediated removal of As(V). When As(III) was the predominant species in the water (Figure 3b), higher HA concentrations resulted in decreased As(III) and increased As(V) levels (*p* < 0.05). Additionally, when both As(V) and As(III) coexisted in the water (Figure 3c), the efficacy of Fe(III) treatment in removing As(V) from aqueous solutions was diminished in the presence of HA, as opposed to when As(V) was present in isolation.

### 3.4. Correlation Analysis of HA Concentrations and As(V) or As(III) Concentrations in Fenton-like Treatment

**Figure 4:**
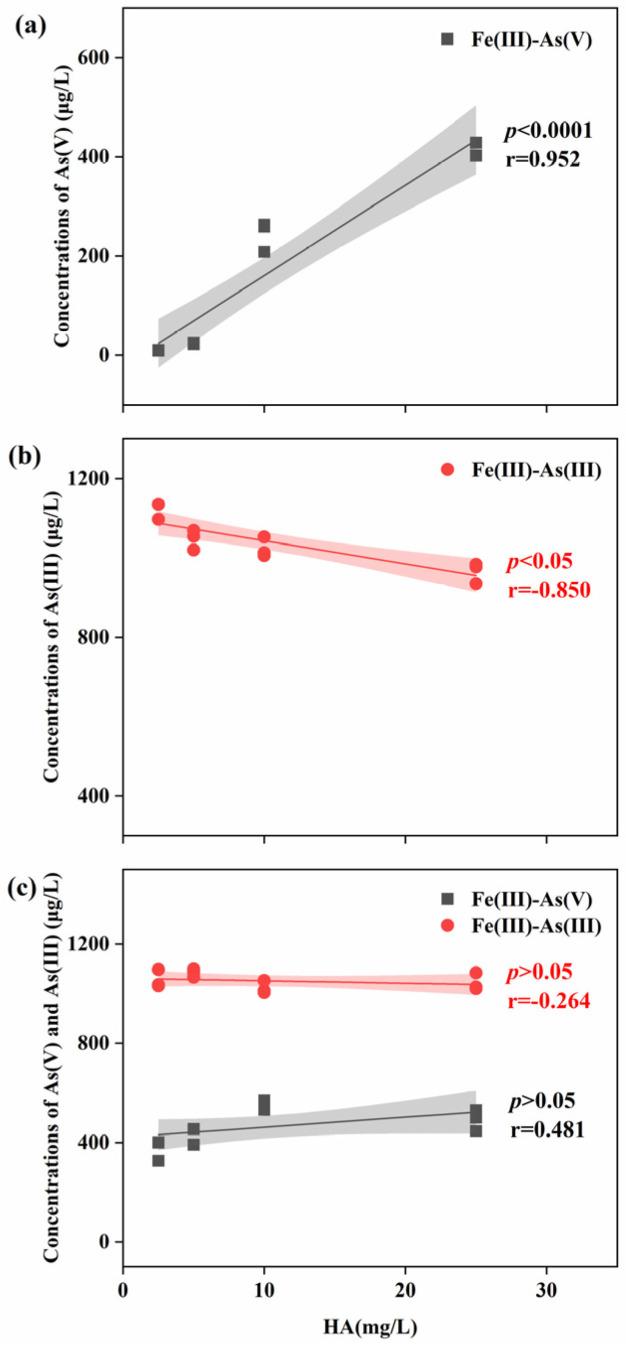
Correlation between HA concentration and As(V)/As(III) concentrations in (**a**) As(V), (**b**) As(III), and (**c**) As(V) and As(III).

Further analysis of the correlation between varying HA concentrations and As(V)/As(III) concentrations under Fenton-like treatment is shown in Figure 4. In the presence of As(V) alone, HA was significantly and positively correlated with As(V) concentration under Fe(III) treatment, indicating that HA reduced the ability of Fe(III) to remove As(V) from water (Figure 4a). When only As(III) was present, HA concentrations under Fe(III) treatment were significantly and negatively correlated with As(III) concentrations. HA promoted the oxidation of As(III) under Fe(III) treatment, with the effect becoming more pronounced at higher HA concentrations (Figure 4b). However, when both As(III) and As(V) were present together, no significant differences in the concentrations of either species were observed (Figure 4c).

### 3.5. Effect of Fenton and Fenton-like Treatment on TOC Removal, pH, and Redox Potential

**Figure 5:**
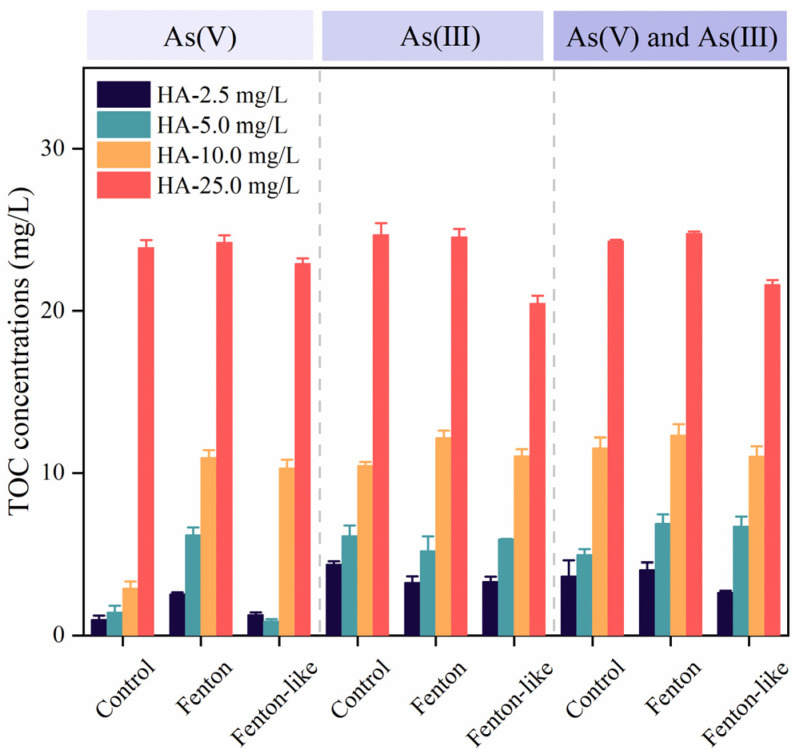
Effect of different treatments on TOC concentration (represented by changes in HA levels): control, Fenton, and Fenton-like. Dashed lines referred to three experimental conditions—1 mg/L As(V) alone, 1 mg/L As(III) alone, and a mixed solution containing 1 mg/L of both As(III) and As(V).

The TOC concentrations in the water after 1 h of reaction are shown in Figure 5. When As(V) was the only species present, the TOC concentrations remained low within the HA concentration range of 2.5 to 10.0 mg/L. However, when As(III) or a combination of As(V) and As(III) were present, the TOC concentrations were higher than in the As(V)-only scenario. At an HA concentration of 25.0 mg/L, the changes in TOC levels for water containing As(III) or both As(V) and As(III) exhibited similar trends. These results suggest that low concentrations of HA facilitate the removal of As(V) and enhance the effect of the Fenton reaction on As species. In contrast, high concentrations of HA (25.0 mg/L) have a less pronounced role in the Fenton reaction or Fe(III) removal.

**Figure 6:**
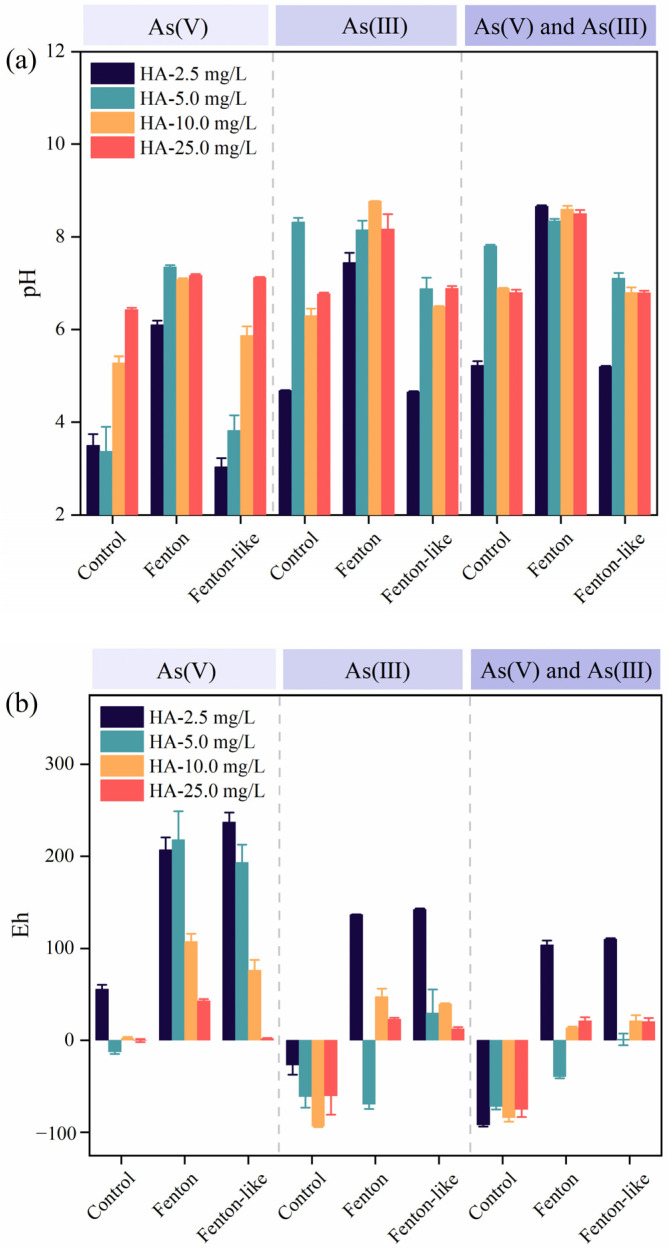
Effect of different treatments on changes in pH (**a**) and Eh (**b**) in water across varying concentrations of HA in Fenton-driven experiments: control, Fenton, and Fenton-like. Dashed lines referred to three experimental conditions—1 mg/L As(V) alone, 1 mg/L As(III) alone, and a mixed solution containing 1 mg/L of both As(III) and As(V).

Further analysis of pH and Eh in the reaction system (Figure 6a,b) revealed that, with the addition of HA concentrations ranging from 2.5 to 25.0 mg/L in natural water, the Fenton reaction driven by H_2_O_2_ and the effectiveness of Fe(III) in As removal were closely linked to pH and Eh levels. The Fenton treatment group exhibited a higher pH level compared to both the control group and the Fe(III) treatment group (Figure 6a). The pH of each treatment group gradually increased as HA concentrations rose from 2.5 to 25.0 mg/L. Additionally, when As(III) was the only species present, or when both As(V) and As(III) coexisted, the pH was higher than in the water containing only As(V) (Figure 6a). Conversely, the Eh was lower under these conditions compared to the scenario with only As(V) present (Figure 6b).

## 4. Discussion

### 4.1. The Effect of a Rainwater-Borne H_2_O_2_-Induced Fenton Process on Changes in As Species in the Presence of HA

The observed dual effect of HA concentration on As removal through Fenton reactions highlights the intricate balance between organic matter and metal ion interactions in aquatic systems. At lower HA concentrations (2.5 to 10 mg/L, Figure 1), the increased reactivity of hydroxyl radicals generated from the Fenton reaction significantly enhanced the oxidation of As(III) to As(V) [26]. This transformation is crucial because As(V) has a higher affinity for adsorption and co-precipitation onto various solid phases, facilitating its removal from aqueous solutions [27,28]. However, this enhancing effect becomes less noticeable at the higher HA concentration of 25 mg/L, indicating that further increases in HA concentration have a limited influence on the reaction. HA has been shown to promote As immobilization through ligand exchange mechanisms and the formation of stable As-Fe-HA and As-HA complexes with iron and As oxidation products, thereby diminishing the efficiency of As(V) removal via the Fenton reaction [29,30,31,32]. The enhanced oxidation of As(III) to As(V) at lower HA concentrations emphasizes the potential for optimizing organic matter levels to improve As remediation strategies.

It has been reported that the optimal pH for the Fenton reaction is approximately 3 [33]; however, HA extends the effective pH range for the Fenton reaction (Figure 6a) by significantly enhancing the oxidation rate of organic compounds in a catalytic Fenton system, even at pH levels between 5 and 7 [34]. Furthermore, the results indicate that the HA-mediated Fenton system is effective for compounds with a high sorption affinity for HA (Figure 1), aligning with previous findings [35]. Further examination of pH and Eh in the reaction system revealed that the efficiency of As species removal was closely related to these factors. pH directly influences the protonation state of functional groups in HA, affecting HA’s aggregation behavior and interactions with As species [36]. In the studied pH range, As(III) primarily exists as the neutral hydroxo complex As(OH)_3_, while As(V) is found in anionic forms, specifically as HAsO_4_^2−^ and H_2_AsO_4_^−^ [22]. Moreover, the redox potential of the HA-As system can vary depending on factors such as the source of HA, its molecular weight, and specific reaction conditions [37]. These findings emphasize the importance of precise control over pH and Eh when optimizing the Fenton reaction for As removal.

### 4.2. Effect of the Fe(III) Products in the Rainwater-Borne H_2_O_2_-Induced Fenton Process on Changes in As Species in the Presence of HA

Fe(III) plays an important role in HA-mediated Fenton processes by promoting the redox cycling between Fe(III) and Fe(II), which is essential for sustaining the Fenton reaction. Fe(II) reacts with H_2_O_2_ to generate additional •OH radicals, thereby enhancing the oxidative capacity of the system [38]. Moreover, HA can influence this process by providing electron-donating sites, facilitating the reduction of Fe(III) back to Fe(II) [39]. This interaction can increase the overall efficiency of the Fenton reaction. However, at higher HA concentrations (10–25 mg/L), the ability of Fe(III) to remove As(V) from water is significantly diminished (Figure 3). HA may enhance the mobility of As(V), reducing Fe(III)’s efficiency in As removal, possibly due to competitive complexation between As(V) and HA [40]. Additionally, Ritter et al. [24] found that Fe(III) can act as a bridge, facilitating the binding of As(V) to Fe(III)-NOM colloids, which plays a crucial role in As transport in the environment. The molecular characteristics of HA, such as molecular weight and functional groups, significantly influence the performance of Fe(III) in the Fenton system. For example, HA from different sources may contain varying concentrations of carboxyl and phenolic groups, which interact with Fe(III), altering its availability and reactivity [41]. Depending on the reaction conditions, these interactions can either enhance or inhibit the catalytic activity of Fe(III).

The presence of Fe(III) in HA-mediated Fenton reactions is integral to the degradation of pollutants, as it facilitates hydroxyl radical generation and redox cycling, thus enhancing the overall efficiency of the process. Understanding the interactions between Fe(III), HA, and environmental factors is crucial for optimizing Fenton-based As treatment systems.

## 5. Conclusions

In this study, a batch experiment was carried out to investigate the variations in As species in the presence of either As(V) or As(III), as well as in their coexistence with HA. The above findings indicate that low concentrations of HA (2.5–10 mg/L) facilitated the oxidation of As(III) and enhanced the removal of As(V) when these As species were present separately. Conversely, at elevated concentrations, the removal efficiency of As(V) is notably diminished. However, the presence of HA hindered the removal of As(V) driven by the Fenton process when both As(V) and As(III) were present together. Furthermore, the presence of HA impeded the Fenton-like removal of As(V), a product of the Fenton reaction, which may be attributed to the competition between As(III) and the HA for the available hydroxyl radical in the Fenton reaction systems. The current study highlights that the presence of high concentrations of HA in water significantly impairs the capacity to eliminate As from the aqueous phase. The findings obtained from the current experiments have advanced understanding of the potential role of rainwater-borne H_2_O_2_ in removing As in open water environments. While these results offer preliminary evidence of HA’s influence on the Fenton reaction’s effectiveness, further research is necessary to explore additional variables and a broader range of environmental conditions to fully optimize this technology.

## Data Availability

The data that support the findings of this study are available from the corresponding author upon reasonable request.

## Supplementary Materials

The following supporting information can be downloaded at: https://www.mdpi.com/article/10.3390/toxics12120845/s1, Table S1: Instrumental operating conditions for the HPLC-ICP-MS system.
