# Supplementary material for: Effect of Fenton-Based Processes on Arsenic Removal in the Presence of Humic Acid
Source: Toxics. 2024 Nov 25;12(12):845. doi: 10.3390/toxics12120845 (PMC11679137; doi:10.3390/toxics12120845)
Supplement: Supplementary file 1 [file toxics-12-00845-s001.zip › toxics-3282395-supplementary.pdf]

Supplementary Material

Table S1. Instrumental operating conditions for the HPLC-ICP-MS system

Parameter	Detailed information
HPLC	Agilent1260
Column	Athena C18-WP column (4.6*250 mm, 5 μm, CNW)<br>CNW guard column (Athena C18-WP, 4.0*20 mm, 5 μm)
Mobile phase	2.5 mM Citric acid/2.5 mM Sodium sulfonate (pH 4.5)
Flow rate	1.0 mL/min
Injected volume	20 μL
ICP-MS	Agilent 7700
RF (forward and reflected power)	1550 W
Spray chamber	Quartz dual channel type
Carrier gas	0.75 L/min
Make-up gas	0.40 L/min
Sample introduction	Meinhard nebulizer
Channels monitored	75, 77 and 78
